# Direct comparison of the in vitro and in vivo stability of DFO, DFO* and DFOcyclo* for ^89^Zr-immunoPET

**DOI:** 10.1007/s00259-019-04343-2

**Published:** 2019-06-03

**Authors:** René Raavé, Gerwin Sandker, Pierre Adumeau, Christian Borch Jacobsen, Floriane Mangin, Michel Meyer, Mathieu Moreau, Claire Bernhard, Laurène Da Costa, Adrien Dubois, Victor Goncalves, Magnus Gustafsson, Mark Rijpkema, Otto Boerman, Jean-Claude Chambron, Sandra Heskamp, Franck Denat

**Affiliations:** 1 Department of Radiology and Nuclear Medicine, Radboudumc, Radboud Institute for Molecular Life Sciences, Nijmegen, The Netherlands; 2 Institut de Chimie Moléculaire de l’Université de Bourgogne, UMR 6302, CNRS, Université Bourgogne Franche-Comté, 9 avenue A. Savary, 21078 Dijon Cedex, France; 3 Global Research Technologies, Novo Nordisk A/S, Novo Nordisk Park, DK-2760 Måløv, Denmark; 4 Institut de Chimie de Strasbourg, UMR 7177, CNRS, Université de Strasbourg, 1 rue Blaise Pascal, 67008 Strasbourg Cedex, France

**Keywords:** ^89^Zr, immunoPET, DFOcyclo*, DFO*, DFO, Monoclonal antibodies

## Abstract

**Purpose:**

Currently, the most commonly used chelator for labelling antibodies with ^89^Zr for immunoPET is desferrioxamine B (DFO). However, preclinical studies have shown that the limited in vivo stability of the ^89^Zr-DFO complex results in release of ^89^Zr, which accumulates in mineral bone. Here we report a novel chelator DFOcyclo*, a preorganized extended DFO derivative that enables octacoordination of the ^89^Zr radiometal. The aim was to compare the in vitro and in vivo stability of [^89^Zr]Zr-DFOcyclo*, [^89^Zr]Zr-DFO* and [^89^Zr]Zr-DFO.

**Methods:**

The stability of ^89^Zr-labelled chelators alone and after conjugation to trastuzumab was evaluated in human plasma and PBS, and in the presence of excess EDTA or DFO. The immunoreactive fraction, IC_50_, and internalization rate of the conjugates were evaluated using HER2-expressing SKOV-3 cells. The in vivo distribution was investigated in mice with subcutaneous HER2^+^ SKOV-3 or HER2^−^ MDA-MB-231 xenografts by PET/CT imaging and quantitative ex vivo tissue analyses 7 days after injection.

**Results:**

^89^Zr-labelled DFO, DFO* and DFOcyclo* were stable in human plasma for up to 7 days. In competition with EDTA, DFO* and DFOcyclo* showed higher stability than DFO. In competition with excess DFO, DFOcyclo*-trastuzumab was significantly more stable than the corresponding DFO and DFO* conjugates (*p* < 0.001). Cell binding and internalization were similar for the three conjugates. In in vivo studies, HER2^+^ SKOV-3 tumour-bearing mice showed significantly lower bone uptake (*p* < 0.001) 168 h after injection with [^89^Zr]Zr-DFOcyclo*-trastuzumab (femur 1.5 ± 0.3%ID/g, knee 2.1 ± 0.4%ID/g) or [^89^Zr]Zr-DFO*-trastuzumab (femur 2.0 ± 0.3%ID/g, knee 2.68 ± 0.4%ID/g) than after injection with [^89^Zr]Zr-DFO-trastuzumab (femur 4.5 ± 0.6%ID/g, knee 7.8 ± 0.6%ID/g). Blood levels, tumour uptake and uptake in other organs were not significantly different at 168 h after injection. HER2^−^ MDA-MB-231 tumour-bearing mice showed significantly lower tumour uptake (*p* < 0.001) after injection with [^89^Zr]Zr-DFOcyclo*-trastuzumab (16.2 ± 10.1%ID/g) and [^89^Zr]Zr-DFO-trastuzumab (19.6 ± 3.2%ID/g) than HER2^+^ SKOV-3 tumour-bearing mice (72.1 ± 14.6%ID/g and 93.1 ± 20.9%ID/g, respectively), while bone uptake was similar.

**Conclusion:**

^89^Zr-labelled DFOcyclo* and DFOcyclo*-trastuzumab showed higher in vitro and in vivo stability than the current commonly used ^89^Zr-DFO-trastuzumab. DFOcyclo* is a promising candidate to become the new clinically used standard chelator for ^89^Zr immunoPET.

**Electronic supplementary material:**

The online version of this article (10.1007/s00259-019-04343-2) contains supplementary material, which is available to authorized users.

## Introduction

^89^Zr has shown great potential as a radionuclide for immunoPET imaging with monoclonal antibodies. ^89^Zr has favourable characteristics: its half-life of 78.4 h matches the kinetics of IgG molecules, it is a residualizing radionuclide that provides images with high target to background ratios [1], and high PET image resolution is obtained because of the relatively low-energy positrons (*E*_mean_ = 395 keV), which is not affected by its γ-emission at 909 keV. Because of these advantages it is frequently used in preference to other long-lived positron emitters, such as ^124^I.

Despite these favourable characteristics for immunoPET, the use of ^89^Zr has a major drawback when used in combination with the clinically approved chelator currently used for antibody labelling, desferrioxamine B (DFO). In vivo studies have revealed that a fraction of ^89^Zr is released from DFO, resulting in accumulation of ^89^Zr in mineral bone [2–6]. This may hamper the detection of bone metastases, an undesired radiation dose to bone marrow, and overestimation of the radiation dose to the red marrow in radionuclide therapy dose planning. Release of ^89^Zr from DFO is the result of suboptimal complexation. ^89^Zr^4+^ demands octacoordination to form stable complexes, while the three hydroxamate groups of DFO only offer hexacoordination, resulting in additional binding of water molecules to saturate the coordination sphere of ^89^Zr^4+^ [5]. Eventually this can result in the release of ^89^Zr, which may subsequently accumulate in mineral bone [5, 7].

To overcome the limited stability of the [^89^Zr]Zr-DFO complex, alternative chelators for [^89^Zr]Zr^4+^ have been developed [8–10]. Examples are DFO derivatives such as DFO* [11] and other “extended” DFO analogues [12], or DFO squaramide ester [13]. Macrocyclic compounds with three or four hydroxamic acid groups [14–16], and chelating agents based on hydroxypyridinone, terephthalamide or catecholate groups as alternatives to hydroxamate-based chelators have also been evaluated [17–24]. Furthermore, the well-known chelator 1,4,7,10-tetraazacyclododecane-1,4,7,10-tetraacetic acid (H_4_DOTA) has also been found to chelate [^89^Zr]Zr^4+^ very efficiently. However, in the absence of an improved, clinically applicable chelator, there is room for more efficient [^89^Zr]Zr^4+^ chelators.

In a quest for a more stable Zr^4+^ chelate, we have developed a new bifunctional DFO derivative, DFOcyclo*-*p*Phe-NCS. By addition of a fourth hydroxamic acid cyclic group, an octadentate DFO derivative with the expected Zr^4+^ coordination properties was synthesized. The in vitro stability of radiolabelled DFOcyclo* was compared with that of radiolabelled DFO and DFO*. Subsequently, all three chelators were conjugated to trastuzumab (Herceptin) by random isothiocyanate-based conjugation, and the in vivo stability of the conjugates was evaluated in tumour-bearing nude mice. Finally, in an attempt to elucidate the mechanism of ^89^Zr release from DFO, the hypothesis that ^89^Zr is released from its chelate upon internalization by cancer cells was tested using a HER2-negative tumour model.

## Materials and methods

### Reagents and equipment specifications

All chemicals, unless otherwise noted, were purchased from Sigma-Aldrich, and used as received without further purification. Ultrapure water produced by a PURELAB Ultra system from ELGA was used throughout (18.2 MΩ cm). NMR spectra were acquired on a Bruker Avance III HD spectrometer operating at 600 MHz. The conjugates were purified by FPLC on an Äkta Pure 25 M chromatography system (GE Healthcare Life Sciences). MALDI-TOF mass spectra were acquired on an ultrafleXtreme instrument (Bruker Daltonics). Size-exclusion chromatography (SEC-HPLC) analyses were performed on a JASCO HPLC system LC-2000 analytical series equipped with a Superdex 200 5/150 GL column. Instant thin-layer chromatography (iTLC) was performed using sheets impregnated with salicylic acid (iTLC-SA; Agilent) eluted with 0.1 M EDTA, pH 5.0, and analysed on an AR-2000 radio-TLC plate reader (Bioscan Inc.). PET/CT scans were acquired on an Inveon animal PET scanner (Siemens Preclinical Solutions).

### Synthesis of chelators

DFO-*p*Phe-NCS was provided by Chematech. DFO* was synthesized according to a previously described procedure [25], with slight modifications enabling isolation of the desired compound in good yield without the need for cumbersome purifications. Likewise, the preparation of DFO*-*p*Phe-NCS was adapted from a previously described procedure [25]. DFOcyclo*-*p*Phe-NCS will soon become commercially available, but in the meantime samples can be obtained from the authors upon request. Briefly, DFOcyclo*-*p*Phe-NCS was obtained by hemisynthesis in three steps from commercially obtained Desferal and a functionalized 1-hydroxypiperidine-2-one (1,2-PIPOH) derivative [26]. After benzyl deprotection under H_2_ on Pd/C, the functionalized tetrachelate was reacted in a final step with a large excess of *p*-phenylene diisothiocyanate (PDITC), and the reaction was stopped at an early stage to favour single condensation. DFOcyclo*-*p*Phe-NCS was obtained in 14% isolated yield after direct injection of the crude reaction mixture onto a semipreparative HPLC column and elution with water/acetonitrile/formic acid with a gradient of 70:30:0.1 to 30:70:0.1. The compound was fully characterized and identified by ^1^H NMR spectroscopy (Supplementary Figs. 1–3), HR-ESI-MS (Supplementary Figs. 4–6), and analytical HPLC (Supplementary Fig. 6).

### ^89^Zr radiolabelling of chelates

To 25 μL 1 M HEPES (pH 7.4) was added 2.5 μL of a 1 mM solution of chelator in water. To this solution was added 5 MBq of [^89^Zr][Zr(oxalate)_4_]^4−^ adjusted to pH 7.4 with 2 M Na_2_CO_3_. The mixture was incubated at room temperature for 15 min. The progress of the reaction was then assayed using iTLC, and complete chelation was observed.

### Stability of the chelates in human plasma and EDTA challenge

The radiochemical purity and the stability of the ^89^Zr-labelled chelates was assessed in plasma and in the presence of EDTA, either by adding 1.5 MBq of radiolabelled chelate to 200 μL of human plasma or by adding 1,000 equivalents of EDTA to 1 MBq of the radiolabelled chelate diluted to 50 μL with PBS. The mixtures were incubated at 37 °C. Aliquots were analysed by iTLC-SA after 24, 48, 72, 96 and 168 h. All samples were prepared in triplicate.

### Determination of the partition coefficient of the radiolabelled chelates

The log *D* values of the radiolabelled chelates (in their –NH_2_ version) were determined using an *n*-octanol/PBS (pH 7.4) system. The radiolabelled chelate (10 μL, 0.5 MBq) was added to 1 mL of PBS, pH 7.4. This solution was supplemented with 1 mL of *n*-octanol previously saturated with water. The mixture was vigorously stirred for 20 min at 25 °C. The two layers were then separated by centrifugation and 50 μL of each layer was collected and counted in an automatic Wizard γ-counter (PerkinElmer). All samples were measured in triplicate. The partition coefficient *D* was calculated using the following formula: log *D* = log (*A*_oct_/*A*_PBS_), where *A*_oct_ and *A*_PBS_ are the activity of the *n*-octanol and PBS layer, respectively.

### Trastuzumab conjugation

**Fig. 1:**
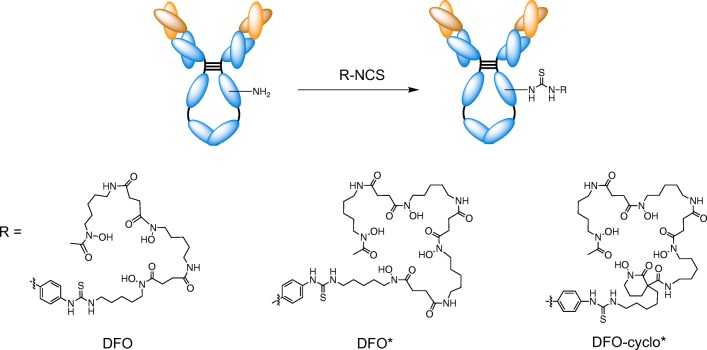
Molecular structures of the conjugates considered in this work: DFO-trastuzumab, DFO*-trastuzumab, and DFOcyclo*-trastuzumab

Bifunctional chelator (30 equivalents, 51.5 μL of a 20 mM stock solution in DMSO) was added to 5.0 mg of purified trastuzumab (in solution at 4 mg/mL in 0.2 M sodium bicarbonate buffer (pH 8.4 buffer for DFO-*p*Phe-NCS and DFOcyclo*-*p*Phe-NCS conjugation) and 0.1 M borate buffer (pH 9.5 buffer for DFO*-*p*Phe-NCS conjugation; Fig. 1). The reaction mixture was stirred at 900 rpm overnight at 37 °C. The mixture was purified by FPLC. Excess chelator was washed away with phosphate buffer (20 mM, pH 7.3) and the conjugate was eluted with 50 mM acetate buffer (pH 3.0), and subsequently the buffer was changed to PBS, pH 7.4, in an ultracentrifugal filter device (Amicon Ultra 2 mL, Ultracel cut-off 30 kDa; Merck Millipore). The protein recovery rate was >89%.

### ^89^Zr radiolabelling of conjugates

To 100 μL of 1 M HEPES, pH 7.3, were added 500 μg of trastuzumab conjugate in PBS, pH 7.4. To this solution were added 25 MBq of a [^89^Zr][Zr(oxalate)_4_]^4−^ solution adjusted to pH 7.4 with 2 M Na_2_CO_3_. The mixture was incubated at room temperature for 30 min. The progress of the reaction was assayed using iTLC, and complete chelation was observed. The radiolabelled conjugate was purified and concentrated using centrifugal filtration units with a 30 kDa molecular weight cut-off filter (Amicon Ultra 0.5 mL; Centrifugal Filtration Units, Merck Millipore). Prior to in vivo injection, ^89^Zr-labelled trastuzumab conjugates were purified by elution on prewashed PD-10 desalting columns (GE Healthcare Life Sciences) eluted with PBS (pH 7.4), instead of purification and concentration using centrifugal filtration units.

### Stability studies of conjugates

#### EDTA challenge of the radiolabelled conjugates

The stability of the conjugates in the presence of EDTA was assessed by adding 1,000 equivalents of EDTA to 0.3–0.5 MBq of the conjugate and diluting the mixture to 50 μL with PBS. The mixture was incubated at 37 °C. Aliquots were analysed by iTLC-SA after 24, 48, 72, 96 and 168 h. All samples were prepared in triplicate.

#### DFO challenge of the radiolabelled conjugates

To assess the stability of the conjugates in the presence of DFO, 2–2.5 MBq of the conjugate was supplemented with 1,000 equivalents of DFO and diluted to 50 μL with PBS. The mixture was incubated at 37 °C. Aliquots were analysed by size-exclusion chromatography after 1, 4, 8, 12, 24 and 48 h. All samples were prepared in triplicate.

### Cell culture

The HER2^+^ human ovarian cancer cell line SKOV-3 (HTB-77; ATCC) and the HER2^−^ human breast cancer cell line MDA-MB-231 (HTB-26; ATCC) were cultured in RPMI-1640 medium (Gibco, ThermoFisher Scientific, Waltham, MA, USA) supplemented with 2 mM glutamine (Gibco) and 10% fetal calf serum (Sigma-Aldrich Chemie BV), at 37 °C in a humidified atmosphere containing 5% CO_2_. Cells were dissociated when 80–90% confluency was reached using 0.05% trypsin (w/v) in 0.53 mM EDTA (Life Technologies) and maintained as proliferating cultures. Mycoplasma contamination was evaluated every 4 months using a MycoAlert mycoplasma detection kit (Lonza, Basel, Switzerland). After thawing, cells were maintained in culture for a maximum of 6 months.

### Binding and internalization assay

The binding and internalization kinetics of trastuzumab conjugated with DFO, DFO* and DFOcyclo* labelled with ^89^Zr were tested using SKOV-3 cells and MDA-MB-231 cells. Cells (0.5 × 10^6^ cells/well) were cultured in six-well plates and incubated with 250 pM ^89^Zr-labelled DFO-trastuzumab, DFO*-trastuzumab or DFOcyclo*-trastuzumab in 0.5% BSA/RPMI-1640 medium (w/v) for 1, 4 and 24 h. Nonspecific binding and internalization was determined by coincubation with 500 nM unlabelled trastuzumab. The membrane-bound fraction was removed from the cells by incubation in an ice-cold solution containing 0.1 M acetic acid and 154 mM NaCl, pH 2.6, for 10 min. Cells were harvested by incubation in 0.1 M NaOH for 5 min. Binding and internalization were determined by counting the radioactivity using a gamma counter (Wizard, PerkinElmer).

### Immunoreactive fraction

The immunoreactive fraction of ^89^Zr-labelled trastuzumab was determined as described by Lindmo et al. [27]. In brief, a serial dilution of SKOV-3 cells ranging from 2.5 × 10^6^ to 1.56 × 10^5^ cells in 0.5 ml were incubated with 250 pM ^89^Zr-labelled DFO-trastuzumab, DFO*-trastuzumab, or DFOcyclo*-trastuzumab at 37 °C for 30 min. Nonspecific binding was determined by incubation of 1.56 × 10^6^ cells with an excess of nonlabelled trastuzumab (500 nM). After incubation, cells were washed twice and the radioactivity in the cell pellet was measured using a gamma counter (Wizard, PerkinElmer).

### In vivo stability studies

#### Animals

The Dutch Central Committee on Animal Research and the local Ethics Committee on Animal Research of Radboud University approved this study under protocol 2015-0071. All animal experiments were performed according to institutional guidelines. Upon arrival, female BALB/cAnNRj-Foxn1^nu^/Foxn1^nu^ mice at 6–8 weeks of age (Janvier Labs, France) were randomly tattooed for identification and were acclimatized for ≥4 days before any experimental procedure. Mice had unlimited access to food and water and were maintained with five or six mice per cage in a controlled environment (22 ± 1 °C, 55 ± 10% humidity, 12-h dark/light cycle). Cages were replaced weekly with clean cages. Mice were assessed daily for welfare and tumour sizes were measured twice a week after tumour inoculation. Mice were randomly allocated to the experimental groups according to a random sequence generator and the biotechnicians performing the experiments were blinded to the group allocation. Tumours were inoculated by subcutaneous (s.c.) injection of 5 × 10^6^ SKOV-3 or MDA-MB-231 cells mixed 2:1 or 1:1 in Matrigel (BD Biosciences; Pharmingen), respectively. Experiments were started when tumours had reached a size of 0.4–0.5 mm^3^.

#### In vivo stability studies

In a first experiment, the in vivo stability of [^89^Zr]Zr-DFOcyclo*-trastuzumab was compared with that of [^89^Zr]Zr-DFO-trastuzumab. SKOV-3 tumour-bearing mice were injected with 200 μL (750 kBq) [^89^Zr]Zr-DFO-trastuzumab and [^89^Zr]Zr-DFOcyclo*-trastuzumab, together with 85 μg nonradiolabelled trastuzumab (Herceptin, Roche) to a total of 100 μg trastuzumab. The ex vivo biodistribution of the ^89^Zr-labelled conjugates was determined by killing the mice by CO_2_/O_2_ asphyxiation at 24 h (five mice per group), 72 h (five mice) and 168 h (six mice) after injection, and collecting tumour, blood, muscle, heart, lung, spleen, pancreas, kidney, liver, stomach, duodenum, colon, salivary glands, sternum, femur, knee and bone marrow. To determine the blood kinetics, blood samples were collected at 1, 3, 24, 72 and 168 h after injection via a tail vein (live mice) or by heart puncture (killed mice). ^89^Zr radioactivity was quantified in the collected samples using a gamma counter (Wizard, PerkinElmer) and expressed as percentage of injected dose per gram of tissue (%ID/g), calculated from the amount of radioactivity measured in aliquots of the injected dose. Simultaneously, mice bearing HER2^−^ MDA-MB-231 s.c. tumours (six mice per group) were also injected with 200 μL (750 kBq) [^89^Zr]Zr-DFO-trastuzumab or [^89^Zr]Zr-DFOcyclo*-trastuzumab to investigate whether [^89^Zr]Zr^4+^ is released from its chelate upon HER2-mediated internalization by cancer cells. Blood kinetics and ex vivo biodistribution analyses were performed as described above.

In a second experiment, the in vivo stability of [^89^Zr]Zr-DFO-trastuzumab and [^89^Zr]Zr-DFOcyclo*-trastuzumab was compared with that of [^89^Zr]Zr-DFO*-trastuzumab, a promising recently described [^89^Zr]Zr^4+^ chelate [25]. SKOV-3 s.c. tumour-bearing mice were injected with 200 μL (5 MBq) [^89^Zr]Zr-DFO-trastuzumab (five mice per group), [^89^Zr]Zr-DFOcyclo*-trastuzumab (six mice) and [^89^Zr]Zr-DFO*-trastuzumab (six mice) together with 5, 11 and 10.3 μg of nonradiolabelled trastuzumab to a total of 100 μg trastuzumab for DFO-trastuzumab, DFOcyclo*-trastuzumab and DFO*-trastuzumab, respectively. Two mice from each group were randomly selected for PET imaging. Mice were imaged under general anaesthesia (2–3% isoflurane/O_2_) for 20 min at 24 and 72 h after injection. PET scans were followed by a CT scan for anatomic reference (spatial resolution of 113 μm, 80 kV, and 500 μA). Scans were reconstructed using Inveon Acquisition Workplace software with iterative three-dimensional ordered subsets expectation maximization using a maximum a priori algorithm with shifted Poisson distribution, with the following parameters: matrix 256 × 256 × 161, pixel size 0.4 × 0.4 × 0.8 mm, with a corresponding beta of 0.05 mm. At 168 h after injection, mice were killed and imaged for 30 min. Subsequently, the biodistribution of the three ^89^Zr-labelled conjugates was determined ex vivo as described before. Blood kinetics were determined in live mice as described above at 1, 3, 24 and 72 h after injection.

### Statistical analysis

Statistical analysis was performed using GraphPad Prism version 5.03 for Windows. Differences in ^89^Zr tissue uptake were tested for significance using Student’s *t*-test or one-way ANOVA with a Bonferroni’s post-hoc test.

## Results

### Chelator structure

DFOcyclo* is an analogue of DFO*. Both chelators contain four hydroxamate moieties, instead of only three for DFO. The main difference between DFO* and DFOcyclo* is the cyclic structure of the additional hydroxamic acid group derived from 1,2-PIPOH. While open-chain hydroxamic acids, including DFO, are known to exist as a mixture of two *E* (*trans*) and *Z* (*cis*) isomers [28–30], the *Z* configuration of the hydroxamate group is imparted by the cyclic structure of PIPO^-^, and is obviously more favourable for Zr^4+^ coordination and could therefore result in a more stable complex [31]. The crystal structure of a Zr^4+^ complex in which the metal is coordinated by four 1,2-PIPO^-^ chelates has been reported previously [32].

The chemical structure of DFOcyclo*-*p*Phe-NCS, the immediate precursor of the DFOcyclo*-trastuzumab bioconjugate, was established by NMR (Supplementary Figs. 1–3) and HR-ESI-MS spectroscopy (Supplementary Figs. 4–5). The ^1^H NMR spectrum in DMSO-*d*_6_ showed the characteristic signals of DFO, the PIPOH additional chelate, and the isothiocyanate-functionalized tether (Supplementary Fig. 1). Noticeably, all the heteroatom-bound hydroxamic (H1, H15, H26, H37), amide (H9, H20, H31) and thiourea (H44, H45) protons could be located in the ^1^H NMR spectrum. The hydroxamic protons and the thiourea proton H45 appeared as broad singlets in the low-field region of the spectrum (>9.5 ppm in DMSO-*d*_6_), while the aliphatic amide protons appeared at 7.5–8 ppm as well-resolved triplets, together with the doublets of the aromatic protons (H46, H47). As shown by the ROESY map, the hydroxamic protons exchanged with water protons, and showed multiple correlations with their neighbours (Supplementary Fig. 2). This information was very valuable for the assignment of the signals of the aliphatic protons, which showed multiple overlaps. In this way, aliphatic protons of the PIPOH fragment (H4, H5, H6), of the DFO backbone (H10–H14, H17, H18, H21–H25, H28, H29, H32–H36, H39), and of the aliphatic tether (H7, H40–H43) could be assigned without any ambiguity (Supplementary Fig. 3).

### Partition coefficient of the radiolabelled chelates

There was no significant difference in the octanol/water partition coefficient between the [^89^Zr]Zr-DFO-NH_2_ and the [^89^Zr]Zr-DFO*-NH_2_ chelates (log *D* −3.61 ± 0.21 and −3.54 ± 0.16, respectively). However, DFOcyclo* exhibited a slightly more lipophilic behaviour than DFO and DFO* (log *D* −2.14 ± 0.10 for [^89^Zr]Zr-DFOcyclo*-NH_2_) due to the addition of the piperidine ring.

### Conjugation trastuzumab substitution ratio

The degree of labelling of the conjugates DFO-trastuzumab, DFOcyclo*-trastuzumab and DFO*-trastuzumab was determined by mass spectrometry. The values obtained ranged from 2.6 to 4.7 for DFO-trastuzumab, and were 3.7 and 2.6 for DFOcyclo*-trastuzumab and DFO*-trastuzumab, respectively.

### Chelate/conjugate labelling, specific activity and stability

**Fig. 2:**
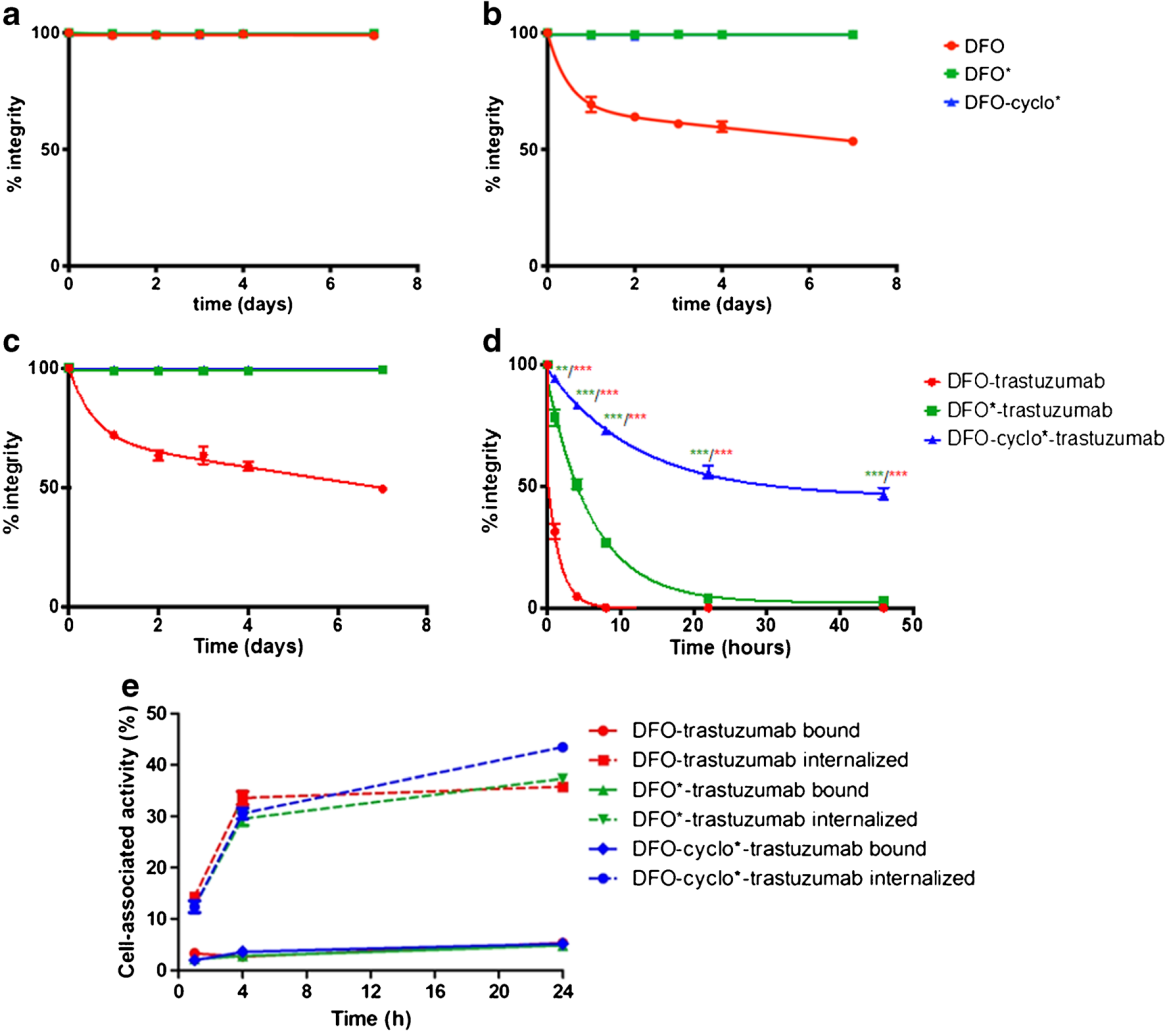
Stability of [^89^Zr]Zr-DFO (*red*), [^89^Zr]Zr-DFO* (*green*) and [^89^Zr]Zr-DFOcyclo* (*blue*) in human plasma (**a**) and in the presence of 1,000 equivalents of EDTA (**b**) at 37 °C. Stability of [^89^Zr]Zr-DFO-trastuzumab (*red*), [^89^Zr]Zr-DFO*-trastuzumab (*green*) and [^89^Zr]Zr-DFOcyclo*-trastuzumab (*blue*) in the presence of 1,000 equivalents of EDTA (**c**) and 1,000 equivalents of DFO (**d**) at 37 °C. Bound and internalized conjugates in HER2^+^ SKOV-3 cells after 1, 4 and 24 h of incubation (**e**). *Error bars* represent standard deviations. **p < 0.01, ***p < 0.001

The chelators were quantitatively labelled with specific activities reaching 4 MBq/nmol. All chelates remained stable in plasma after 7 days (Fig. 2a). Importantly, [^89^Zr]Zr-DFO* and [^89^Zr]Zr-DFOcyclo* showed higher stability than [^89^Zr]Zr-DFO in the presence of excess EDTA. The integrity of [^89^Zr]Zr-DFO decreased to 53%, while the [^89^Zr]Zr-DFO* and [^89^Zr]Zr-DFOcyclo* complexes remained >98% intact after 7 days (Fig. 2b).

The conjugates were quantitatively labelled at a specific activity of 50 GBq/g (7.5 MBq/nmol). Size-exclusion chromatography revealed the presence of aggregates in small amounts for the three radioconjugates: 4.7 ± 0.3%, 3.6 ± 0.2% and 6.5 ± 0.7% for [^89^Zr]Zr-DFOcyclo*-trastuzumab, [^89^Zr]Zr-DFO-trastuzumab and [^89^Zr]Zr-DFO*-trastuzumab, respectively. This aggregation was probably because of the choice of the bioconjugation moiety, as phenyl-isothiocyanate is known to cause aggregation [33, 34]. Like the free chelates, the ^89^Zr-labelled DFO*- and DFOcyclo*-based conjugates showed higher stability than the DFO-based conjugate. The competition experiments with EDTA gave results similar to those with the chelates alone (Fig. 2c). However, incubation of the radiolabelled conjugates with DFO clearly discriminated the stability of the three conjugates (Fig. 2d). While the integrity of [^89^Zr]Zr-DFO*-trastuzumab decreased to 50% in 4 h, [^89^Zr]Zr-DFOcyclo*-trastuzumab reached 50% of integrity in 48 h, and seemed to plateau around 50% integrity, suggesting a much higher stability for this radiolabelled conjugate. The DFO-based conjugate exhibited the lowest stability, with an almost total release of radionuclide in less than 10 h.

### In vitro assays

The cell binding and internalization assay using HER2^+^ SKOV-3 cells showed 2–4% binding for the three conjugates at 1 and 4 h, and approximately 4–5% at 24 h (Fig. 2e). Internalization of the three conjugates in HER2^+^ SKOV-3 cells was about 12–14% at 1 h, and increased to approximately 30–40% at 4 and 24 h (Fig. 2e). Incubation with an excess of nonradiolabelled trastuzumab or incubation with HER2^−^ MDA-MB-231 cells resulted in binding and internalization of the three conjugates of <1% at all time points (Supplementary Fig. 7). The immunoreactive fraction of all three conjugates was >90%.

### Pharmacokinetics and biodistribution of the radiolabelled conjugates in HER2^+^ tumour-bearing mice

**Fig. 3:**
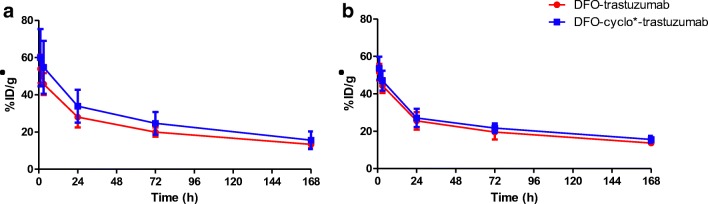
Blood kinetics of [^89^Zr]Zr-DFO-trastuzumab (*red*) and [^89^Zr]Zr-DFOcyclo*-trastuzumab (*blue*) in HER2^+^ SKOV-3 tumour-bearing mice (**a**) and HER2^−^ MDA-MB-231 tumour-bearing mice (**b**). *Error bars* represent standard deviations

**Fig. 4:**
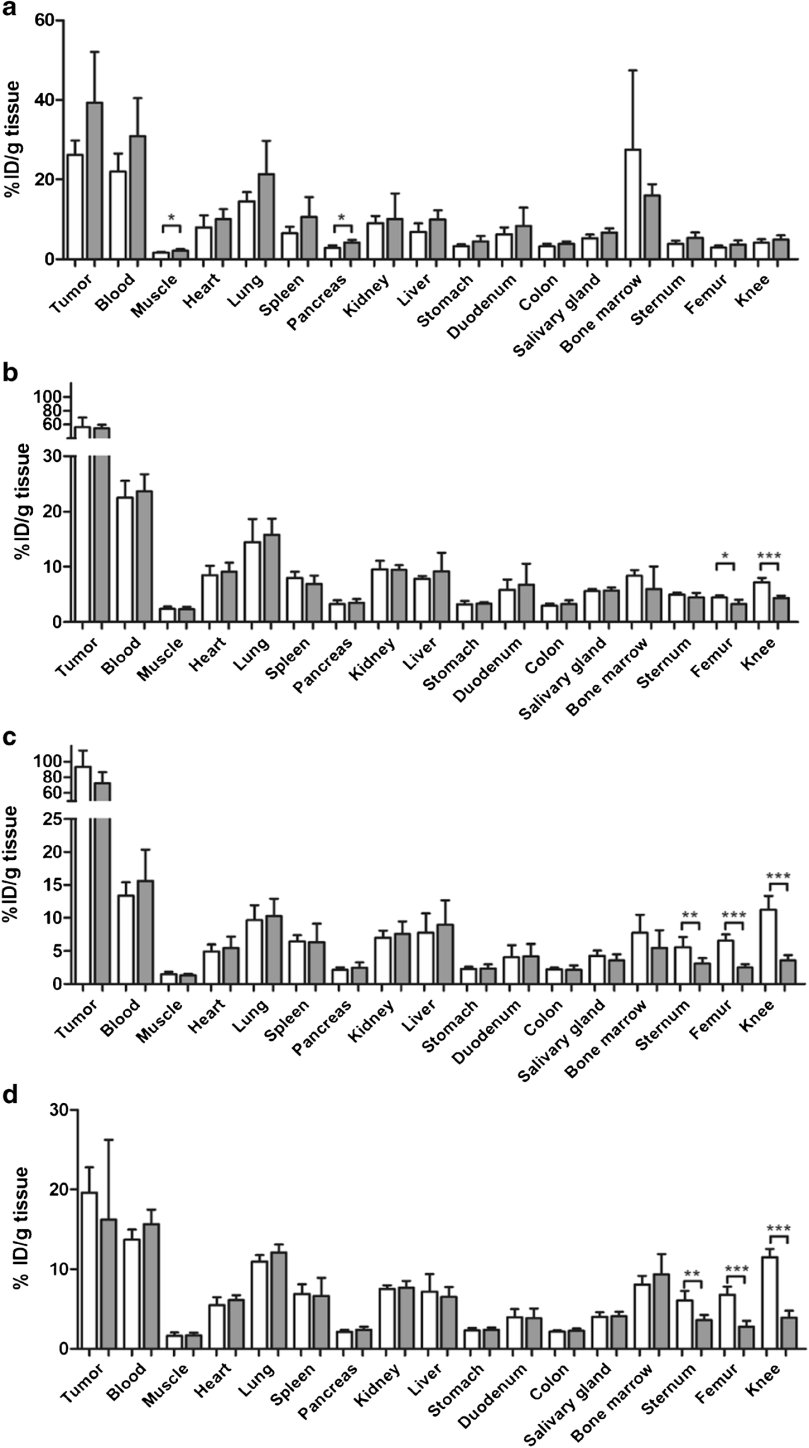
Biodistribution of [^89^Zr]Zr-DFO-trastuzumab (*white bars*) and [^89^Zr]Zr-DFOcyclo*-trastuzumab (*grey bars*) in HER2^+^ SKOV-3 tumour-bearing mice (**a–c**) at 24 h (**a**), 72 h (**b**) and 168 h (**c**) after injection, and in HER2^−^ MDA-MB-231 tumour-bearing mice (**d**) at 168 h after injection. *Bars* represent means with standard deviations. *p < 0.05, **p < 0.01, ***p < 0.001

The in vivo characteristics of [^89^Zr]Zr-DFO-trastuzumab and [^89^Zr]Zr-DFOcyclo*-trastuzumab were determined by intravenous injection into HER2^+^ SKOV-3 s.c. tumour-bearing nude mice. Blood levels determined at 1, 3, 24, 72 and 168 h after injection showed similar patterns for both conjugates (Fig. 3a). Tumour uptake increased from 26.2 ± 3.6%ID/g and 39.4 ± 12.7%ID/g at 24 h after injection to 93.1 ± 20.9%ID/g and 72.1 ± 14.6%ID/g at 168 h after injection for [^89^Zr]Zr-DFO-trastuzumab and [^89^Zr]Zr-DFOcyclo*-trastuzumab, respectively, but was not significantly different between the conjugates (Fig. 4a–c). Bone uptake was significantly lower in mice injected with [^89^Zr]Zr-DFOcyclo*-trastuzumab at 72 h after administration (femur *p* < 0.05, knee *p* < 0.001; Fig. 4b). At 168 h after injection, ^89^Zr uptake was significantly lower in the sternum (*p* < 0.01), femur (*p* < 0.0001) and knee (*p* < 0.0001) in mice injected with [^89^Zr]Zr-DFOcyclo*-trastuzumab than in mice injected with [^89^Zr]Zr-DFO-trastuzumab. Uptake in muscle and pancreas was significantly higher in mice injected with ^89^Zr-DFOcyclo*-trastuzumab at 24 h after injection (*p* < 0.05), but was not different at 72 and 168 h after injection (Fig. 4a–c). Uptake in other organs was not significantly different for the two conjugates.

### In vivo blood kinetics and biodistribution in HER2^−^ tumour-bearing mice

To investigate the hypothesis that ^89^Zr is released from the chelator after cellular internalization, two groups of mice bearing HER2^−^ MDA-MB-231 s.c. tumours were injected with 750 kBq (100 μg) [^89^Zr]Zr-DFO-trastuzumab or [^89^Zr]Zr-DFOcyclo*-trastuzumab. Blood levels determined at 1, 3, 24, 72 and 168 h after injection showed a pattern similar to the kinetics in mice with HER2^+^ tumours (Fig. 3b). Ex vivo biodistribution showed a significantly lower uptake in HER2^−^ tumours than in HER2^+^ tumours for both conjugates (*p* < 0.001; Fig. 4c, d). ^89^Zr uptake in bone (sternum, femur and knee) in HER2^−^ tumour-bearing mice was comparable to that in HER2^+^ tumour-bearing mice: in both groups bone uptake was significantly lower in mice injected with [^89^Zr]Zr-DFOcyclo*-trastuzumab.

### In vivo stability comparison to DFO*

**Fig. 5:**
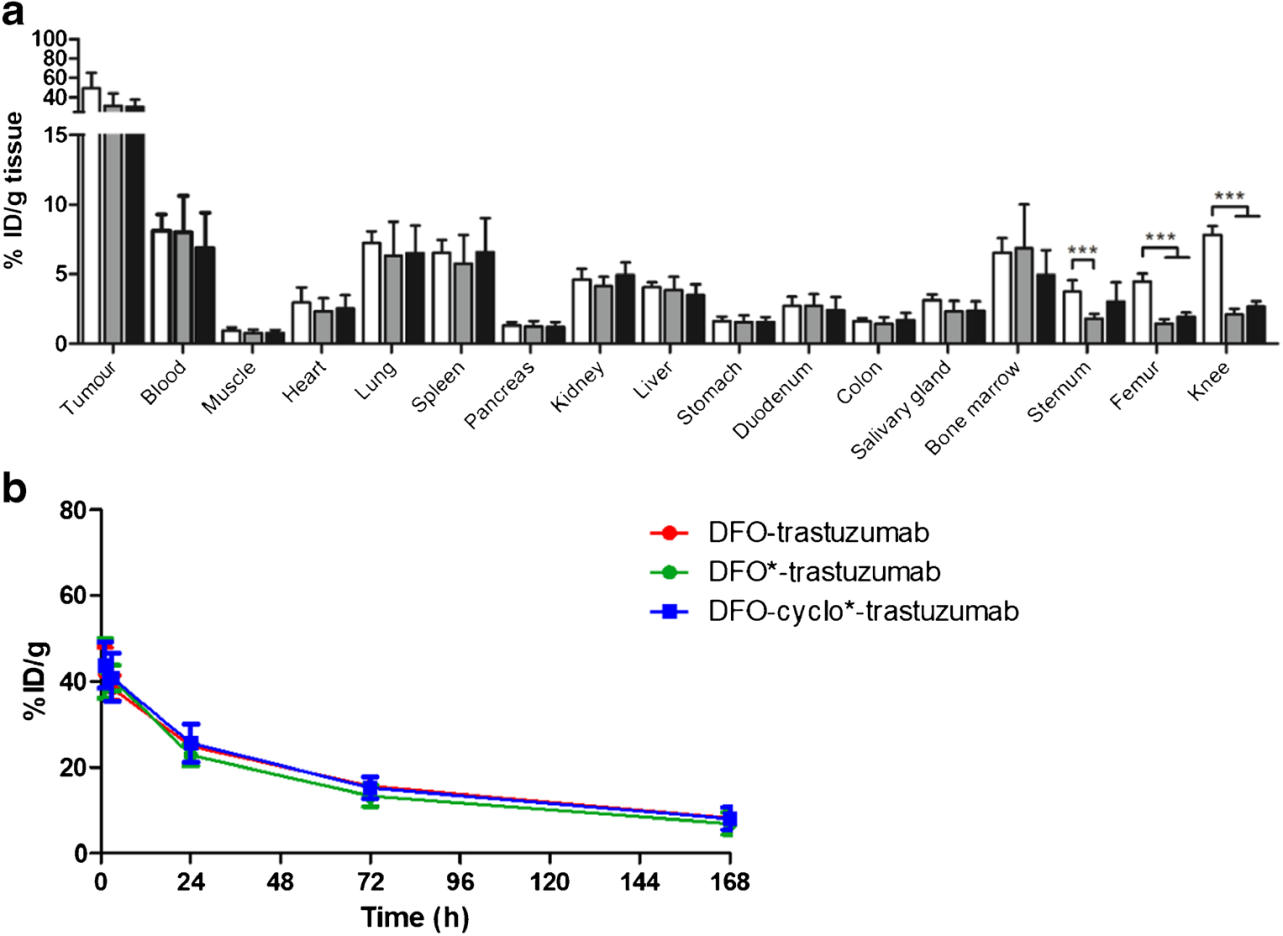
**a** Biodistribution of [^89^Zr]Zr-DFO-trastuzumab (*white bars*), [^89^Zr]Zr-DFOcyclo*-trastuzumab (*grey bars*) and [^89^Zr]Zr-DFO*-trastuzumab (*black bars*) at 168 h after injection and blood kinetics. **b** Blood kinetics after injection in HER2^+^ SKOV-3 tumour-bearing mice. *Bars* represent means with standard deviations. *** *p* < 0.0001

**Fig. 6:**
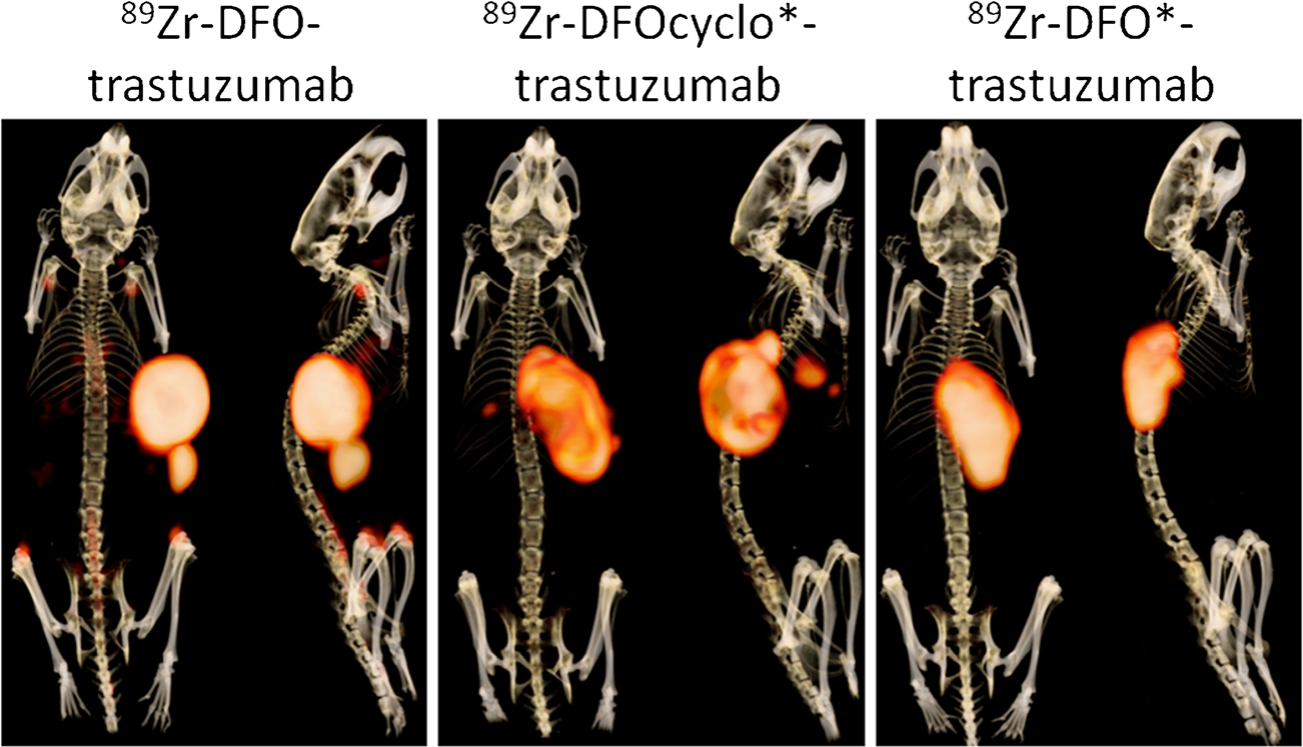
PET images of mice injected with 5 MBq [^89^Zr]Zr-DFO-trastuzumab, [^89^Zr]Zr-DFOcyclo*-trastuzumab and [^89^Zr]Zr-DFO*-trastuzumab imaged at 168 h after injection

In a final experiment, the in vivo stability of [^89^Zr]Zr-DFO-trastuzumab and [^89^Zr]Zr-DFOcyclo*-trastuzumab were compared with that of [^89^Zr]Zr-DFO*-trastuzumab. Tumour uptakes were similar and not significantly different among the three conjugates (Fig. 5a). ^89^Zr uptake in the femur and knee was significantly lower in mice injected with [^89^Zr]Zr-DFOcyclo*-trastuzumab and [^89^Zr]Zr-DFO*-trastuzumab than in mice injected with [^89^Zr]Zr-DFO-trastuzumab (*p* < 0.0001). ^89^Zr uptake in the sternum was significantly lower in mice injected with [^89^Zr]Zr-DFOcyclo*-trastuzumab than in mice injected with [^89^Zr]Zr-DFO-trastuzumab (*p* < 0.0001), but not in mice injected with [^89^Zr]Zr-DFO*-trastuzumab. Blood kinetics and uptake in all other tissues were similar among the three conjugates (Fig. 5b). Most importantly, PET images revealed bone uptake in mice injected with [^89^Zr]Zr-DFO-trastuzumab, but not in mice injected with [^89^Zr]Zr-DFOcyclo*-trastuzumab or [^89^Zr]Zr-DFO*-trastuzumab at 168 h after injection (Fig. 6).

## Discussion

We report here a new ^89^Zr chelator, DFOcyclo*, with improved in vitro and in vivo stability characteristics compared with currently the most common chelator used clinically, DFO. Because of the octacoordination obtained by the addition of a fourth cyclic hydroxamic acid group, DFOcyclo* produced a more stable complex with [^89^Zr]Zr^4+^ than the hexacoordinating chelator DFO. The results of competition assays with excess EDTA and DFO emphasized the improved stability of the complex indicated by easy ^89^Zr release from DFO and DFO-trastuzumab, while it was significantly less easily released from DFOcyclo* and DFOcyclo*-trastuzumab. The results of in vivo studies confirmed these findings, as indicated by significantly lower ^89^Zr uptake in mineral bone following injection of ^89^Zr-DFOcyclo*-trastuzumab. In a head-to-head comparison with DFO*, a related tetrahydroxamic DFO derivative with higher stability than the parent DFO as described by Vugts et al. [25], DFO* and DFOcyclo* showed similar in vitro and in vivo stability characteristics. Only when challenged with an excess of DFO in vitro (Fig. 2c) and in terms of sternum uptake in vivo (Fig. 5a), did DFOcyclo* outperform DFO*, suggesting that [^89^Zr]Zr-DFOcyclo* shows higher thermodynamic stability than [^89^Zr]Zr-DFO*. This higher stability can most likely be ascribed to the preorganization effect of the additional cyclic *cis*-hydroxamate binding unit found in DFOcyclo* as compared with the open-chain chelating motifs of DFO*.

In addition to DFO*, other chelating agents such as DFO squaramide ester, hydroxamic acid-based macrocycles, hydroxypyridinone (HOPO) derivatives, and DOTA have been proposed as alternatives to DFO for labelling antibodies with ^89^Zr. The DFO squaramide ester has the advantages of being more soluble in water and causing less aggregation of antibodies, and shows promising stability reflected by lower in vivo ^89^Zr bone uptake at 24 h and 48 h after injection than DFO [13]. However, at a later time point (96 h after injection), the difference was not so pronounced, while in our study the difference in ^89^Zr bone uptake between mice injected with [^89^Zr]Zr-DFOcyclo*-trastuzumab and [^89^Zr]Zr-DFO-trastuzumab increased over time, suggesting that [^89^Zr]Zr-DFOcyclo* has higher in vivo stability than [^89^Zr]Zr-DFO squaramide.

Besides DFO derivatives, macrocyclic chelators have also been tested as ^89^Zr immunoPET agents. Zhai et al. and Summer et al. evaluated fusarinine C, a cyclic siderophore containing three hydroxamic acid groups, conjugated to a cyclic RGD peptide, and an EGFR-binding Affibody, respectively [15, 35]. While only offering hexacoordination for ^89^Zr complexation, in vitro and in vivo results showed promising stability characteristics. Again, in their studies the follow-up time (24 h) was too short for direct comparison of the results with those of our study, because we observed differences in bone uptake only beyond 72 h after injection. Another macrocyclic hydroxamic acid-based chelator with a hexadentate coordination was evaluated by Boros et al. [16]. Conjugated to trastuzumab, higher bone uptake was observed in mice injected with ^89^Zr-labelled trastuzumab through their newly developed chelate than labelled through DFO, indicating lower in vivo stability than DFO. Ma et al. tested a tripodal tris(hydroxypyridinone) ^89^Zr chelate conjugated to trastuzumab in vivo, but also found increased ^89^Zr bone uptake in mice injected with ^89^Zr-labelled trastuzumab through their new chelator compared to DFO, demonstrating lower in vivo stability [18].

Deri et al. proposed another octadentate ^89^Zr chelator, 3,4,3-(LI-1,2-HOPO), consisting of four 1,2-HOPO units (aromatic counterpart of our 1,2-PIPOH) appended to a spermine backbone (referred to here as LI-HOPO) [19]. In vivo studies indicated that [^89^Zr]Zr-LI-HOPO-trastuzumab has higher stability than [^89^Zr]Zr-DFO-trastuzumab, as reflected by significantly lower bone uptake at later time points (168–336 h after injection). However, [^89^Zr]Zr-DFO-trastuzumab showed a higher tumour-to-blood uptake ratio, indicating that the in vivo HER2-targeting properties of [^89^Zr]Zr-LI-HOPO-trastuzumab may have been compromised. Another [^89^Zr]Zr-HOPO-trastuzumab conjugate, incorporating 2,3-HOPO chelating groups, was described by Tinianow et al. [20], but [^89^Zr]Zr-2,3-HOPO-trastuzumab did not show higher stability than [^89^Zr]Zr-DFO-trastuzumab, as reflected by higher bone uptake in animals injected with [^89^Zr]Zr-2,3-HOPO-trastuzumab. Pandya et al. evaluated the use of H_4_DOTA as a chelator for [^89^Zr]Zr^4+^ [24]. Despite [^89^Zr]Zr-DOTA showing promising higher in vivo stability than [^89^Zr]Zr-DFO, DOTA may not be a suitable chelator for use in ^89^Zr immunoPET, because of the harsh labelling conditions (incubation at 95 °C for 1 h) that would disrupt the integrity of an antibody, requiring labelling before conjugation. Although comparing the results of the present study with those of previous studies investigating alternative ^89^Zr immunoPET chelates is difficult, the present results suggest that [^89^Zr]Zr^4+^ complexes with DFO* and DFOcyclo* show promising in vivo stability characteristics and therefore may be used as alternatives to DFO bioconjugates for ^89^Zr immunoPET, as the latter chelator forms less-stable complexes.

The exact ^89^Zr release mechanism from DFO bioconjugates is not yet fully understood. It has been hypothesized by Holland et al. that ^89^Zr-labelled metabolites enter the circulation after slow intratumoral metabolism [5]. In a study attempting to confirm this hypothesis, HER2^−^ tumour-bearing mice showed bone uptake after injection of ^89^Zr-labelled DFO-trastuzumab or DFOcyclo*-trastuzumab similar to that in HER2^+^ tumour-bearing mice, but about 4.5 times lower tumour uptake. This implies that with a fraction of the tumour uptake, and consequently only a fraction of intratumoral metabolism, ^89^Zr is still being released from the DFO-trastuzumab at the same rate. We therefore hypothesize that ^89^Zr is released from the chelator during physiological metabolism and is excreted via biological pathways rather than undergoing intratumoral metabolism.

### Conclusion

The use of DFO as a chelator for ^89^Zr immunoPET may result in undesired uptake of ^89^Zr in mineral bone. ^89^Zr-labelled DFOcyclo* and DFOcyclo*-trastuzumab showed higher in vitro and in vivo stabilities than DFO and DFO-trastuzumab. DFOcyclo* is a highly promising candidate to become the new clinically used standard chelator for ^89^Zr immunoPET.

## Electronic supplementary material

ESM 1 (DOCX 135 mb)
