# Supplementary material for: Direct comparison of the in vitro and in vivo stability of DFO, DFO* and DFOcyclo* for 89Zr-immunoPET
Source: Eur J Nucl Med Mol Imaging. 2019 Jun 3;46(9):1966–77. doi: 10.1007/s00259-019-04343-2 (PMC6647232; doi:10.1007/s00259-019-04343-2)
Supplement: Supplementary file 1 — (DOCX 135 mb) [file 259_2019_4343_MOESM1_ESM.docx]

**Direct comparison of the in vitro and in vivo stability of DFO, DFO* and DFOcyclo* for ^89^Zr-immunoPET**

René Raavé^1^, Gerwin Sandker^1^, Pierre Adumeau^2^, Christian Borch Jacobsen^3^, Floriane Mangin^2^, Michel Meyer^2^, Mathieu Moreau^2^, Claire Bernhard^2^, Laurène Da Costa^2^, Adrien Dubois^2^, Victor Goncalves^2^, Magnus Gustafsson^3^, Mark Rijpkema^1^, Otto Boerman^1^, Jean-Claude Chambron*^2,4^, Sandra Heskamp*^1^, Franck Denat*^2^

^1^	Radboud Institute for Molecular Life Sciences, department of Radiology and Nuclear Medicine, Radboudumc, Nijmegen, the Netherlands.

^2^	Institut de Chimie Moléculaire de l'Université de Bourgogne, UMR 6302, CNRS, Université Bourgogne Franche-Comté, 9 avenue A. Savary, 21078 Dijon Cedex, France.

^3^	Global Research Technologies, Novo Nordisk A/S, Novo Nordisk Park, DK-2760 Måløv, Denmark.

^4^	Institut de Chimie de Strasbourg, UMR 7177, CNRS, Université de Strasbourg, 1 rue Blaise Pascal, 67008 Strasbourg Cedex, France.

**Supplementary Information**

**Synthesis**

**General**

Chemicals were used as received. HPLC-gradient grade acetonitrile was obtained from Carlo Erba. Aqueous mobile phases for HPLC were prepared using deionized water (18.2 MΩ cm) produced by a PURELAB Ultra system from ELGA. ^1^H NMR spectra were recorded with a Bruker Avance III HD spectrometer operating at 600 MHz. The following abbreviations and their combinations are used: s, singlet; t, triplet; qt, quartet; qn, quintuplet; m, multiplet; br, broad. High-resolution mass spectra (HR-ESI-MS) were obtained with a LTQ Orbitrap XL mass spectrometer from Thermo Scientific equipped with an electrospray ionisation source. Analytical RP-HPLC-MS analyses were performed using an UltiMate 3000 system Dionex (Thermo Scientific) fitted with a DAD detector and coupled to a low-resolution mass spectrometry detector (Thermo Scientific), equipped with an ESI source. Separation was achieved using an RP KinetexTM column (Phenomenex) (2.6 μm, 100 Å, 50 × 2.1 mm) with ultrapure water and HPLC-grade acetonitrile (A: H_2_O 0.1% formic acid and B: MeCN 0.1% formic acid). The purity of DFOcyclo*-*p*Phe-NCS was determined by integration of the HPLC-MS chromatograms at 214 nm. Purifications by semi-preparative RP-HPLC were performed using an Ultimate 3000 system Dionex (Thermo Scientific) equipped with an UV-visible detector, using a BetaBasic-18 column (Thermo Scientific) (5 μm, 150 Å, 150 × 30 mm) at 15 mL/min with HPLC-grade eluents. The following gradient was used: 30% of B in 10 min followed by an increase of B to 70% in 40 min. FPLC purification of the conjugates was performed on an Äkta Pure 25 M chromatography system (GE Healthcare Life Sciences) equipped with a HiTrap® MabSelectTM column (MabSelect resin, Protein A, cross-linked agarose, column I.D. 7 mm, bed dimensions 7 x 25 mm, bed volume 1 mL). Size-exclusion chromatography (SEC-HPLC) analyses were performed on a JASCO HPLC system LC-2000 analytical series equipped with a Superdex® 200 5/150 GL column, running for 10 min at 0.5 mL/min with phosphate buffer saline (pH 7.4) supplemented with 0.1 M EDTA. Instant thin-layer chromatography (iTLC) was performed using salicylic acid impregnated sheets (iTLC-SA, Agilent) eluted with 0.1 M EDTA, pH 5.0, and analyzed on an AR-2000 radio-TLC plate reader (Bioscan Inc.). In the iTLC experiments, free [^89^Zr]Zr^4+^ cations and [^89^Zr][Zr(EDTA)] elute with the solvent front, while radiolabeled DFO-derivatives remain at the origin.

**DFO*-*p*Phe-NCS**

The DFO* was synthesized by a modified procedure of the originally published synthesis.^1^ The synthesis of *N*-(Benzyloxy)-*N*-[5-*N*´-(benzyloxycarbonyl)aminopentyl]carbamic acid tert-butyl ester was accomplished as described in the literature.^2^ The remaining synthetic steps then proceeded as follows:

*N*-(benzyloxy)-*N*-[5-(benzyloxycarbonylamino)pentyl]succinamic acid:

To a vial containing *N*-(benzyloxy)-*N*-[5-*N*´-(benzyloxycarbonyl)aminopentyl]carbamic acid tert-butyl ester (2.0 mmol) was added TFA (6.0 mL) and the reaction stirred at room temperature for 90 min. The TFA was then evaporated as an azeotrope with toluene. To the vial was then added 99% ethanol (10 mL), DABCO (6.0 mmol) and succinic anhydride (6.0 mmol). The mixture was stirred at 55 °C for 30 min. The mixture was acidified with 1.0 M HCl (aq) and the compound extracted with diethyl ether. The solution was dried over MgSO_4_, evaporated, and the desired compound purified by flash chromatography (EtOAc in heptane) in 82% yield as a yellow oil. The NMR and mass spectra were equivalent to the originally published ones.^2^

Protected DFO*:

The coupling of *N*-(benzyloxy)-*N*-[5-(benzyloxycarbonylamino)pentyl]succinamic acid to DFO was achieved as follows:

To vial 1, containing *N*-(benzyloxy)-*N*-[5-(benzyloxycarbonylamino)pentyl]succinamic acid (1.4 mmol) in dichloromethane (35 mL), was added trimethylamine (1.4 mmol) and ethyl chloroformate (1.4 mmol). The reaction was stirred at room temperature for 10 min. To vial 2, containing DFO (1.8 mmol) was added 99% ethanol (50 mL) and trimethylamine (2.0 mmol). The mixture was stirred at room temperature for 10 min. Vial 2 was then poured into vial 1 and the reaction stirred at room temperature for 10 min. After evaporating the solvent, 50 mL of water and 1.0 mL of 1.0 M HCl (aq) were added. The remaining DFO dissolved and the desired product could be recovered by filtration. The water was removed by washing with acetone and then diethyl ether. The desired product was isolated in 64% yield as a crisp white solid. Hydrogenation of protected DFO* over Pd/C in methanol as described in the originally published procedure^1^ afforded DFO*. The DFO*-*p*Phe-NCS was then synthesized as reported earlier.^1^

**DFOcyclo*-*p*Phe-NCS**^3^

DFOcyclo* (0.143 g, 0.18 mmol) was reacted with *p*-phenylene diisothiocyanate (0.349 g, 1.82 mmol) in dry DMF (4 mL) under argon for 3 h at room temperature. The solvent was removed in vacuo and the residue dissolved in DMSO for purification by semi-preparative HPLC using water/acetonitrile/0.1% formic acid as eluent with the gradient 70:30:0.1 to 30:70:0.1. Solvent removal by lyophilization afforded a colorless solid (0.024 g, 14 % yield). ^1^H NMR (600 MHz, DMSO-*d*_6_): *δ* 9.76 (br s, 1 H; N(O)H); 9.63 (br s, 2 H; N(O)H); 9.59 (br s, 2 H; N(O)H); 7.76 (t, *J* = 5.3 Hz, 3 H; N(O)H); 7.45 (AB, *J*_AB_ = 9.0 Hz, Δ*ν* = 109.0 Hz , 4 H; Ar-H); 7.47 (t, *J* = 5.6 Hz, 1 H; N(O)H); 3.53 (m, 10 H); 3.10 (m, 1 H); 3.00 (br qt, 6 H); 2.57 (br t, *J* = 3.6 Hz, 3 H); 2.27 (br t, *J* = 7.6 Hz, 5 H); 1.96 (s, 3 H; CH_3_); 1.81–1.75 (m, 2 H); 1.72–1.62 (m, 2 H); 1.53–1.45 (m, 7 H); 1.38 (qn, *J* = 7.6 Hz, 6 H); 1.29–1.13 (m, 12 H) ppm. HR-ESI-MS: Found, 979.47567; calcd for C_44_H_71_N_10_O_11_S_2_ ([**M** + H]^+^), 979.47397 amu.

**References**

1)	M. Patra, A. Bauman, C. Mari, C. A. Fisher, O. Blacque, D. Häussinger, G. Gasser, T. L. Mindt, *Chem. Commun*. **2014**, *50*, 11523 – 11525.

2)	N. Kadi, D. Oves-Costales, F. Barona-Gomez, G. L. Challis *Nat. Chem. Biol.* **2007**, *3*, 652 – 656.

3)	This compound will become soon commercially available. In the meantime, samples can be obtained upon request.

**Supplementary Figure 1**

^1^H NMR spectrum (DMSO-*d*_6_, 600 MHz) of DFOcyclo*-*p*Phe-NCS. Protons in the 1–4 and 7–10 ppm regions have been integrated separately in order to cancel the effects of differing relaxation times.

**Supplementary Figure 2**

Detail of the ROESY ^1^H/^1^H NMR spectrum (DMSO-*d*_6_, 600 MHz) of DFOcyclo*-*p*Phe-NCS. The map shows correlations between hydroxamic and amide protons on the one hand, and alkyl protons on the other hand.

**Supplementary Figure 3**

Detail of the ROESY ^1^H/^1^H NMR spectrum (DMSO-*d*_6_, 600 MHz) of DFOcyclo*-*p*Phe-NCS. The map shows correlations between alkyl protons.

**Supplementary Figure 4**

HR-ESI-MS of DFOcyclo*-*p*Phe-NCS.

**Supplementary Figure 5**

HR-ESI-MS of DFOcyclo*-*p*Phe-NCS (detail and simulation).

**Supplementary Figure 6**

HPLC chromatogram of DFOcyclo*-*p*Phe-NCS.

**Supplementary Figure 7**

In vitro binding/internalization studies in HER2- negative cells (**a**), and blocking studies with excess unlabeled trastuzumab in HER2+ cells (**b**) and HER2- cells (**c**), and in vitro experiments with HER2- cells.
